# Positive Psychology Interventions as an Opportunity in Arab Countries to Promoting Well-Being

**DOI:** 10.3389/fpsyg.2021.793608

**Published:** 2022-01-05

**Authors:** Asma A. Basurrah, Mohammed Al-Haj Baddar, Zelda Di Blasi

**Affiliations:** ^1^Department of Psychology, Faculty of Arts and Humanities, King Abdulaziz University, Jeddah, Saudi Arabia; ^2^School of Applied Psychology, University College Cork, Cork, Ireland; ^3^Technical Department, Research Triangle Institute International, Amman, Jordan

**Keywords:** positive psychology, positive psychology interventions, well-being, Arab countries, mental health

## Introduction

Extensive research on Positive Psychology Interventions (PPIs)—defined as activities that focus on promoting positive feelings, thoughts, or behaviour (Sin and Lyubomirsky, 2009)—has been found to be generally effective in promoting well-being and reducing mental illness (e.g., Chakhssi et al., 2018; Hendriks et al., 2020). In a recent review of 347 studies, PPIs had a significant effect on promoting quality of life and well-being and reducing anxiety and depression (Carr et al., 2020). While the ratio of non-Western to Western randomised controlled trials (RCTs) evaluating PPIs has improved quite dramatically in the past decade, most of these studies (82%) remain narrow in focus in terms of the culture of their participants, having been developed and tested in Western Educated Industrialised Rich Democratic (WEIRD) countries (Hendriks et al., 2019). Therefore, they may not be as effective in other countries such as the Arab countries where there are various cultural differences. Culture can play a very important role in enhancing PPIs engagement, acceptability, and eventually, effectiveness (Hendriks and Graafsma, 2019).

Arab countries, home to 5% of the world's population, have a burden of mental health problems above global levels (GBD 2015 Eastern Mediterranean Region Mental Health Collaborators, 2018). Prevalent stigma, war and conflict were some of the contributors (Maalouf et al., 2019). Thus, to properly address this cultural stigma against mental health problems, PPIs could provide an additional role alongside traditional psychology approaches (e.g., Cognitive Behavioural therapy - CBT). Further, in light of the political conditions that some Arab countries are going through and the negative effects that they cause, it has become necessary to use the method of *prevention* that is the focus of PP and not only of *treatment* as a strategy for applications and practise. It has been noted that the mental health care system in the Arab region focuses highly on illnesses treatment and neglects the significant role that PP plays in enhancing human potential and well-being. The Arab population is becoming more influenced by many global issues, including the COVID-19 pandemic which is having a negative impact on mental health and well-being (Salari et al., 2020). In response, Waters et al. (2021) have recently argued how positive psychological factors can play a significant role in buffering mental illness, enhancing mental health throughout the pandemic, and building positive processes and capacities that can help to promote future mental health.

In developing indigenous and culturally sensitive PPIs (Lambert et al., 2015) among the Arab region, there are important cultural factors that should be considered. These include spiritual traditions and interdependent cultural concepts including collectivistic ideas of the self, emotions and values which differ quite dramatically from western cultures.

## Arab Culture

The “Arab World,” which comprises 5% of the world's population, refers to Arab countries in the Middle East and North Africa. Despite the increasing pace and progress of different businesses in many Arab countries, individual communities are still limited in certain prominent economic places. Arab people rather gather in collective communities. They generally share the same Arabic Islamic culture, speak the same language, and descend from the same Arab families when going back in history, no matter what nationality they have nowadays. Yet, very many changes came to communities before borders were drawn between countries and after. For instance, geography, topography, demography, and socio-economic status affect peoples' nature of jobs and lifestyles. For example, Arabs in the east, west, or the middle have similar tongues; however, almost every Arab country has its accent. Therefore, people need to go back to the formal Arabic language “Fus-ha” or learn more about different Arabic dialects when communicating cross-countries.

According to Hofstede (2011) based on the individualism-collectivism dimension, the Arab countries (Eastern society) have been classified as a collectivist culture, where their identity and decisions are influenced by social systems. In contrast, the United States (Western society) has been classified as an individualist culture focusing on individual decisions. Although there are degrees of individualism among Arabs in certain countries, they still share collective common characteristics. Here, we explore the meaning of collectivistic conceptions of the self, emotions, values, and religion in Arab countries and how they differ from those in individual Western cultures.

### The Self

Since the field of PP focuses on the development of *self* , it is vital to recognise that self-concept varies across cultures. Individualism generally emphasises the self-directed and autonomous individual (Realo et al., 2002). People in individualist countries focus primarily on their personal characteristics (e.g., motives, abilities) to build their self-concept. On the other hand, collectivism refers to several social structures that highly value the groups to which people belong, such as family and tribe (Realo, 2003). People in collectivist countries such as the Arab countries focus primarily on their relationships with others to build their self-concept (Markus and Kitayama, 1991). Hence, research from Arab countries should focus on the core elements of collectivism when implementing PPIs to make a significant, meaningful impact. For example, people from a collective culture may experience a greater enhancement in well-being when practising interventions that are more prosocial and group-oriented such as compassion, performing acts of kindness, writing a gratitude letter, and using character strengths in everyday context including social context, compared with self-oriented interventions such as *identifying* character strengths.

### Emotions, Value, and Religion

Research shows that Western culture emphasises the goal of maximising positive emotions, while Eastern culture emphasises embracing and balancing positive and negative emotions (Leu et al., 2011). To illustrate, culture plays an important role in influencing perceptions of happiness. People in individualist countries value happiness highly. In contrast, people from collective countries value low arousal positive emotions (Leu et al., 2011) and exhibit a fear of happiness (Joshanloo, 2013; Joshanloo and Weijers, 2019). Hence, the influence of positive emotions plays a limited role in the mental health of Eastern society. Speaking of Value, for an Arab, the family is the centre of honour and the most important social unit. This loyalty has an impact on every part of an Arab's life. Arabs honour their families and highly value their friendships. Therefore, future research could shed more light on group-oriented interventions (e.g., kindness) that focus more on the relationship with family, friends, and community.

When it comes to religion, Arab society has a rich culture in values and beliefs that place a particularly high emphasis on spirituality. Religion is the most important and distinctive aspect of Arab culture. Arab countries vary in terms of religion, with Islam being predominant. Interestingly, the Islamic religion may influence PP. For example, forgiveness “al'afwu,” aligns with Islamic teachings (Warsah, 2020), having strong philosophical and religious roots (Peterson and Seligman, 2004). While this concept is highly recommended in PP to be cultivated for psychological health and well-being (Wulandari and Megawati, 2020), it is also encouraged to be embedded in ourselves, according to Islamic teachings. This confirms how both PP and Islam clearly propose the importance to cultivate forgiveness both towards ourselves and others (Warsah, 2020).

### Well-Being (Hedonic and Eudaimonic)

It is necessary here to clarify what is meant by hedonic and eudaimonic definitions of well-being. While the term “Hedonic” is based on the pursuit of maximum levels of pleasure (feeling good), the term “Eudaimonic,” on the other hand, is based on meaning and the development of virtues (functioning well; Keyes and Annas, 2009). Research indicates that cultures are not equally supportive of hedonic and eudaimonic aspects (Joshanloo and Jarden, 2016). In comparison to collectivism, hedonism appears to be more congruent with individualism (Joshanloo, 2014). Pleasure and positive emotions are considered a way to pursue happiness in Western culture, while this method is not highly favoured in Eastern cultures (Lee et al., 2013); Because they consider suffering and negative emotions as contributing factors to spiritual development. Therefore, the eastern perspective is more in line with a eudaimonistic view which emphasises virtues, meaning, and feeling of belongingness. However, although we believe that both approaches can be found to a certain level in both cultures, the differences suggest that there are different routes to happiness.

## Positive Psychology in The Arab Region

Positive psychology - “the scientific study of what makes life most worth living” (Seligman and Csikszentmihalyi, 2000) - is one of the newest branches of psychology. It focuses on three main pillars: (1) positive subjective experiences (such as happiness and love); (2) positive individual characters (such as gratitude and compassion); and (3) positive institutions (for the application of positive principles within institutions and organizations; Seligman and Csikszentmihalyi, 2000). Some of the main topics of interest in positive psychology include character strengths, gratitude, hope, happiness, mindfulness, optimism, positive thinking, and resilience. Within the applications of this science, various domains of well-being (such as happiness, engagement, positive emotions, and meaning) can be enhanced by practising positive psychology interventions (Sin and Lyubomirsky, 2009). Interestingly, these interventions have also produced benefits beyond well-being, such as reduced mental health issues (Chakhssi et al., 2018; Hendriks et al., 2020).

Nowadays, PP is increasingly noticed in the Arab world (Rao et al., 2015; Lambert and Pasha-Zaidi, 2019). In recent years, there has been a great effort to explore the PP field across Arab countries. Several initiatives have emerged aimed at promoting well-being and flourishing. For example, the Middle East Journal of Positive Psychology published its first volume in 2015. On the International Day of Happiness in 2017, United Arab Emirates University launched its Emirates Center for Happiness Research. Meanwhile, Effat University in Saudi Arabia started the first Positive Psychology and Well-being Research Lab, the first symposium, and the first PP course. In 2019, Louise Lambert and Nausheen Pasha-Zaidi published the first regional text entitled “Positive Psychology in the Middle East/North Africa.” There are other efforts spent in the region as well; such as the work conducted in Egypt. Ibrahim Younus established the Arab Association for Positive Psychology https://www.psycholearn.com/en/associative.php which provides several courses on PP and also published a book entitled “The Power of Positive Psychology” (Younus, 2017).

Despite this development, nothing can compare to the quantity and even quality of research in other countries. In light of our observations, the few PP studies may be attributed to the lack of awareness of the constructive approach and prevention compared to problem-solving. An Arab dentist once said: “people do not come to me until they are badly in pain.” In any case, many studies have taken place in Arab countries in the last decade, more of which are descriptive and less of which experimental. For example, Abdel-Khalek (2010) found positive correlations between quality of life, subjective well-being, and religiosity among students in Kuwait. Abdel-Khalek (2016) developed “the Arabic Scale of Religiosity,” which significantly correlated with PP variables among students in Algeria, Kuwait, and Egypt.

Recently, empirical studies on PPIs have been conducted in Arab-Islamic countries. More attention has been placed on promoting well-being and alleviating the high burden of mental health problems in the region (GBD 2015 Eastern Mediterranean Region Mental Health Collaborators, 2018). For instance, several studies have examined the effectiveness of mindfulness among university students (e.g., Thomas et al., 2016; Al-Ghalib and Salim, 2018; Awad, 2019), parents of children with autism (Rayan and Ahmad, 2016) and addicted adults (Al-Rashidi, 2018). These interventions were found to have positive effects on health and well-being outcomes. Participants who practised mindfulness reported reduction in stress and depression as well as improvement in well-being and resilience. Moreover, the results of a recent pilot study found positive impacts on enhancing emotional regulation and reducing stress among Arab teachers (Berkovich-Ohana et al., 2020). Some studies have also begun to examine various interventions targeting character strengths (Basurrah et al., 2020; Chérif et al., 2021), self-compassion (Elaiwah, 2017), positive thinking (Haddad, 2014; Mohammed et al., 2014), and hope (Zaki, 2016) among adults and university students. Interestingly, a recent study by Lambert et al. (2019) has examined the impact of PPIs on fear of happiness (a belief that happiness or positive emotions can bring forward negative consequences; Joshanloo, 2013) among people from collective countries. Lambert et al. (2019) provided evidence that a 14-week PPIs programme has an impact on reducing the fear and fragility of happiness beliefs among university students in the United Arab Emirates.

Since Arab countries place great emphasis on spirituality, several authors have considered the aspect of religion when implementing PPIs (e.g., Saeedi et al., 2015). For instance, an empirical study by Al-Seheel and Noor (2016) found that expressing gratitude towards God “Allah” increases the happiness of the Muslim more than the usual gratitude intervention. Additionally, Al-Ghalib and Salim (2018) examined a religiously sensitive mindfulness-training programme with university students and found a positive effect on life satisfaction and a slight reduction in stress, depression, and anxiety. Hence, the role of religion is vital to make the interventions more relevant to Arab culture where people can connect Islamic philosophy with PP theories and practises. These studies reviewed here provide further support for the need for culturally sensitive interventions among the Arab population.

More recently, the first systematic review of PPIs in Arab countries was conducted by Basurrah et al. (2021; the protocol has been published in *BMJ Open* and the final manuscript has been submitted for publication). Reviewing a total of 39 RCTs and quasi-experimental studies, the most commonly studied interventions were mindfulness, positive thinking, and resilience. Only a handful of studies examined gratitude, character strengths, forgiveness, self-compassion, savouring, or finding flow. Risk of bias analysis revealed that most studies from Arab countries have several methodological limitations. This included a lack of protocol guidelines, few well-designed randomised controlled trials (RCTs), blinding issues, small sample sizes, lack of active control groups, and lack of research into certain populations (e.g., teachers, employees, and people in distress in refugee camps). These are important methodological issues that need to be considered in future research.

## Discussion

PP was founded on an individualistic framework (Christopher and Hickinbottom, 2008). To ensure that PPIs are culturally meaningful, this paper includes some cultural elements of the Arab region that should be considered to serve the needs of the Arab population. In the Arab world, some people may believe that PP is solely about happiness and being positive. If so, it is important to educate the public about the findings of rigorous studies evaluating PPIs such as gratitude, hope or flow on health, well-being and performance. To explain the value and effectiveness of these evidence-based approaches, examples might include studies linking gratitude with benefits for people with heart disease (Cousin et al., 2021), or how PPIs can promote quality of life in cancer patients (Casellas-Grau et al., 2014).

There is some evidence about PPIs being effective in improving well-being in the Arab region, but research in this region is still in its infancy and little information is available regarding PPIs and the experience of Arabs participating in such interventions. Hence, there is still much more to investigate in this regard. Future research should empirically examine the effectiveness of various unstudied interventions such as savouring, gratitude, self-compassion, character strengths or finding flow. A combination of quantitative and qualitative approaches is required to provide an in-depth understanding of Arabs' experience and impressions of PPIs and how and why they work. This may provide useful information to inform the appropriate design of PPIs to suit Arab culture and needs. In addition, since it has been reported that published positive psychology empirical studies were largely overrepresented by female participants (Rao and Donaldson, 2015; Hendriks et al., 2019), it would be important for future research to consider the effect of gender when developing PPIs.

Furthermore, while some research on PPIs has been carried out in the region, most of these studies have been of poor quality that suffered from small sample sizes, confounding factors, and a high degree of bias. To overcome these issues, future research should improve research quality, including protocol guidelines and well-designed RCTs. In particular, research should include randomisation, allocation, blinding, power analysis to determine adequate sample size, active control groups to reduce bias, and follow-up periods of at least 12 months.

Among the most popular resources that future PP practitioners may refer to are the International Positive Psychology Association (IPPA) and the International Positive Education Network (IPEN). Also, we recommend the Middle East Journal of Positive Psychology and the first regional book (Lambert and Pasha-Zaidi, 2019). To develop and/or adapt a multi-component PPIs, we would recommend referring to Character Strengths (Peterson and Seligman, 2004), the theory of well-being (PERMA Model: Positive Emotion, Engagement, Relationships, Meaning, and Accomplishment; Seligman, 2011), and the Five Ways to Well-being: Connect, Be Active, Take Notice, Keep Learning, and Give (Aked et al., 2008). Finally, single-component PPIs are based on theories like Broaden-And-Build (Fredrickson, 2004), Hope (Snyder et al., 2002), and Self-Determination (Deci and Ryan, 2012) can be also considered.

Two other concepts that would be usefully applied are “Savouring” (Bryant and Veroff, 2007) and Phillip Zimbardo's Time Perspective (Stolarski et al., 2015). For instance, savouring has a positive temporal orientation to reminiscing the good memories from the past, enjoying the hedonic present moments, and willing proactively to make a better future. Now, what is even maybe more interesting for Muslims is “Transcendent Future” where all intentions, words and actions are devoted to that life after death.

In addition, there is a model already prepared for the clinical purpose called Positive Psychotherapy (PPT). Tayyab Rashid and Martin Seligman published a PPT manual for clinicians in 2018. And for cross-cultural applications, including Muslim Arab culture, Rashid and Al-Haj Baddar (2019) have written a paper presenting PPT and its efficacy. PPT overview in detail shows sessions, themes, skills, practise, and cultural considerations where a mixture of PPIs based on PERMA, Strengths, and other PP concepts can be found.

Regarding PP measurements, it is highly recommended to generate indigenous tools based on cultural backgrounds reviewed here in the article. An example of this is a tool for measuring Muslim well-being by including domains beyond what is found in Western literature. So far, in the Arab region, several attempts have been made to adapt PP tools. For example, Marei Salama-Younes (Salama-Younes and Massoud, 2018) validated the Arabic versions of different measures of well-being (e.g., satisfaction with life scale, the subjective vitality scale). Other validated scales as well-include the self-compassion scale (Alabdulaziz et al., 2020), and the passion scale (Salama-Younes and Hashim, 2018).

## Conclusion

This paper has discussed the cultural aspects of Arab countries and the need of developing culturally sensitive PPIs. The past decade has seen an increase in mental health problems in the Arab region. Stigma and lack of awareness have been always there. Among the issues are also wars, conflicts, and displacement in many Arab countries such as Iraq, Lebanon, and Palestine (Hassan et al., 2016). These indicate an urgent need to prevent mental illness and promote well-being. Indeed, we highlighted the importance of integrating culture with PPIs, following the guidelines for the cultural adaptation of PPIs (Hendriks and Graafsma, 2019) and the ethical guidelines for PP practise (Jarden et al., 2020). Another significant contribution of this paper is to highlight the importance of improving the quality of research conducted in Arab countries. In brief, this paper has several practical implications. It is an open invitation for researchers, policymakers, and practitioners alike to be more exposed to PP; as well as to better conceptualising and adapting interventions and measurement tools to the local culture in different sectors to maximise their effectiveness. And as we consider it as an invitation for us too, here we are starting a PPIs practise guide in Arabic language and for Arab practitioners. The insights gained from this paper may be useful for placing well-being on top of priorities for achieving individual, organisational, and optimal national functioning in different sectors. There is also a need to change the stigma or negative beliefs about seeking health for psychological support, which is an issue for countries in the Middle East (Baess, 2019). It is hoped that this article will encourage researchers and practitioners to investigate and integrate culturally sensitive positive psychology interventions and practises so that people of Arab cultures can benefit from these.

## Author Contributions

AB, MA-H, and ZD participated in the conception of the article. AB and MA-H drafted the article with support from ZD. All authors contributed to the final version of the manuscript, critically revised the article, provided feedback, and approved the final version to be published.

## Conflict of Interest

The authors declare that the research was conducted in the absence of any commercial or financial relationships that could be construed as a potential conflict of interest. The handling editor declared a past collaboration with one of the authors ZD.

## Publisher's Note

All claims expressed in this article are solely those of the authors and do not necessarily represent those of their affiliated organizations, or those of the publisher, the editors and the reviewers. Any product that may be evaluated in this article, or claim that may be made by its manufacturer, is not guaranteed or endorsed by the publisher.

## References

[B1] Abdel-Khalek A. M.. (2010). Quality of life, subjective wellbeing, and religiosity in Muslim college students. Qual. Life Res. 19, 1133–1143. 10.1007/s11136-010-9676-7 20585988

[B2] Abdel-Khalek A. M.. (2016). The Arabic Scale of Religiosity: its development, psychometric, parameters, and relation with positive psychology variables. J. Psychol. Stud. Egypt 26, 159–182 Retrieved from https://search.mandumah.com/Record/806407 (in Arabic).

[B3] Aked J. Marks N. Cordon C. Thompson S. (2008). Five Ways to Wellbeing: A Report Presented to the Foresight Project on Communicating the Evidence Base for Improving People's Well-Being. London: New Economics Foundation.

[B4] Alabdulaziz H. Alquwez N. Almazan J. U. Albougami A. Alshammari F. Cruz J. P. (2020). The Self-Compassion Scale Arabic version for baccalaureate nursing students: a validation study. Nurse Educ. Today 89:104420. 10.1016/j.nedt.2020.104420 32276172

[B5] Al-Ghalib S. J. Salim A. Y. (2018). A mindfulness-based intervention to enhance university student wellbeing in Saudi Arabia. Middle East J. Posit. Psychol. 4, 142–157. Retrieved from https://middleeastjournalofpositivepsychology.org/index.php/mejpp/article/view/70

[B6] Al-Rashidi M.. (2018). The Effectiveness of Transcendental Meditation for Mindfulness Improvement and Stress Reduction Among Drug Addicts and Psychotropic Substances at Al Amal Mental Health Center in Riyadh (doctoral dissertation). King Khaled University, Saudi Arabia (in Arabic).

[B7] Al-Seheel A. Y. Noor N. M. (2016). Effects of an Islamic-based gratitude strategy on Muslim students' level of happiness. Mental Health Religion Cult. 19, 686–703. 10.1080/13674676.2016.1229287

[B8] Awad M.. (2019). The Effectiveness of Counselling Mindfulness-Based Program in Improving Psychological Resilience Among University Students (Master's thesis). Amnahour University, Egypt (in Arabic).

[B9] Baess K.. (2019). Engaging in Psychological Interventions: A Middle Eastern Perspective. The University of Manchester, United Kingdom.

[B10] Basurrah A. Lambert L. Setti A. Murphy M. Warren M. Shrestha T.. (2021). Effects of positive psychology interventions in Arab countries: a protocol for a systematic review. BMJ Open 11:e052477. 10.1136/bmjopen-2021-052477 PMC8323366 34326058

[B11] Basurrah A. A. O'Sullivan D. Chan J. S. (2020). A character strengths intervention for happiness and depression in Saudi Arabia: a replication of Seligman et al.'s (2005) study. Middle East J Posit. Psychol. 6, 41–72. Retrieved from https://www.middleeastjournalofpositivepsychology.org/index.php/mejpp/article/view/100

[B12] Berkovich-Ohana A. Lavy S. Shanboor K. (2020). Effects of a mindfulness intervention among Arab teachers are mediated by decentering: a pilot study. Front. Psychol. 11:2475. 10.3389/fpsyg.2020.542986 33132951 PMC7550638

[B13] Bryant F. B. Veroff J. (2007). Savoring: A New Model of Positive Experience. Mahwah, NJ: Lawrence Erlbaum Associates, Publishers.

[B14] Carr A. Cullen K. Keeney C. Canning C. Mooney O. Chinseallaigh E.. (2020). Effectiveness of positive psychology interventions: a systematic review and meta-analysis. J. Positive Psychol. 16, 1–21. 10.1080/17439760.2020.1818807

[B15] Casellas-Grau A. Font A. Vives J. (2014). Positive psychology interventions in breast cancer. A systematic review. Psychooncology 23, 9–19. 10.1002/pon.3353 23897834

[B16] Chakhssi F. Kraiss J. T. Sommers-Spijkerman M. Bohlmeijer E. T. (2018). The effect of positive psychology interventions on well-being and distress in clinical samples with psychiatric or somatic disorders: a systematic review and meta-analysis. BMC Psychiatry 18, 1–17. 10.1186/s12888-018-1739-2 29945603 PMC6020379

[B17] Chérif L. Wood V. M. Watier C. (2021). Testing the effectiveness of a strengths-based intervention targeting all 24 strengths: results from a randomized controlled trial. Psychol. Rep. 124, 1174–1183. 10.1177/0033294120937441 32597373

[B18] Christopher J. C. Hickinbottom S. (2008). Positive psychology, ethnocentrism, and the disguised ideology of individualism. Theory Psychol. 18, 563–589. 10.1177/0959354308093396

[B19] Cousin L. Redwine L. Bricker C. Kip K. Buck H. (2021). Effect of gratitude on cardiovascular health outcomes: a state-of-the-science review. J. Positive Psychol. 16, 348–355. 10.1080/17439760.2020.1716054

[B20] Deci E. L. Ryan R. M. (2012). Self-determination theory, in Handbook of Theories of Social Psychology, eds P. A. M. Van Lange, A. W. Kruglanski, and E. T. Higgins (Sage Publications Ltd.), 416–436.

[B21] Elaiwah S.. (2017). The effectiveness of a counselling program of self-compassion to improve psychological resilience among university students. J. Coll. Educ. 68, 113–183. 10.21608/MKMGT.2017.133337

[B22] Fredrickson B. L.. (2004). The broaden–and–build theory of positive emotions. Philos. Transact. R. Soc. London Ser. B Biol. Sci. 359, 1367–1377. 10.1098/rstb.2004.1512 15347528 PMC1693418

[B23] GBD 2015 Eastern Mediterranean Region Mental Health Collaborators (2018). The burden of mental disorders in the Eastern Mediterranean region, 1990-2015: findings from the global burden of disease 2015 study. Int. J. Public Health 63(Suppl. 1), 25–37. 10.1007/s00038-017-1006-1 28776247 PMC5973970

[B24] Haddad A.. (2014). The Effectiveness of a Training Program Based on Positive Thinking to Reduce Test Anxiety Among a Sample of Tenth-Grade Students. (doctoral dissertation). Yarmouk University, Jordan (in Arabic).

[B25] Hassan G. Ventevogel P. Jefee-Bahloul H. Barkil-Oteo A. Kirmayer L. J. (2016). Mental health and psychosocial wellbeing of Syrians affected by armed conflict. Epidemiol. Psychiatric Sci. 25, 129–114. 10.1017/S2045796016000044 26829998 PMC6998596

[B26] Hendriks T. Graafsma T. (2019). Guidelines for the cultural adaptation of positive psychology interventions. *Caribbean J. Psychol*. 11, 7–32. Available online at: https://www.uwipress.com/cjp-vol-11-i1-a1/

[B27] Hendriks T. Schotanus-Dijkstra M. Hassankhan A. De Jong J. Bohlmeijer E. (2020). The efficacy of multi-component positive psychology interventions: a systematic review and meta-analysis of randomized controlled trials. J. Happ. Stud. 21, 357–390. 10.1007/s10902-019-00082-1

[B28] Hendriks T. Warren M. A. Schotanus-Dijkstra M. Hassankhan A. Graafsma T. Bohlmeijer E.. (2019). How WEIRD are positive psychology interventions? A bibliometric analysis of randomized controlled trials on the science of well-being. J. Positive Psychol. 14, 489–501. 10.1080/17439760.2018.1484941

[B29] Hofstede G.. (2011). Dimensionalizing cultures: the Hofstede model in context. Online Read Psychol. Cult. 2, 2307–0919. 10.9707/2307-0919.1014

[B30] Jarden A. Rashid T. Roache A. Lomas T. Al-Ahmadi A. (2020). Ethical guidelines for positive psychology practice (version 1.0: Arabic). Int. J. Wellbeing 9, 1–38. 10.5502/ijw.v9i3.9218486778

[B31] Joshanloo M.. (2013). The influence of fear of happiness beliefs on responses to the satisfaction with life scale. Person. Ind. Differ. 54, 647–651. 10.1016/j.paid.2012.11.011

[B32] Joshanloo M.. (2014). Eastern conceptualizations of happiness: fundamental differences with western views. J. Happ. Stud. 15, 475–493. 10.1007/s10902-013-9431-1

[B33] Joshanloo M. Jarden A. (2016). Individualism as the moderator of the relationship between hedonism and happiness: a study in 19 nations. Person. Ind. Differ. 94, 149–152. 10.1016/j.paid.2016.01.025

[B34] Joshanloo M. Weijers D. (2019). Islamic perspectives on wellbeing, in Positive Psychology in the Middle East/North Africa, eds L. Lambert, and N. Pasha-Zaidi (Cham: Springer), 237–256.

[B35] Keyes C. L. M. Annas J. (2009). Feeling good and functioning well: Distinctive concepts in ancient philosophy and contemporary science. J. Positive Psychol. 4, 197–201. 10.1080/17439760902844228

[B36] Lambert L. Pasha-Zaidi N. (eds.). (2019). Positive Psychology in the Middle East/North Africa: Research, Policy, and Practise. Cham: Springer.

[B37] Lambert L. Pasha-Zaidi N. Passmore H.-A. York Al-Karam C. (2015). Developing an indigenous positive psychology in the United Arab Emirates. Middle East J. Positive Psychol. 1, 1–23. 10.1057/9781137558237_832649257

[B38] Lambert L. Passmore H. A. Joshanloo M. (2019). A positive psychology intervention program in a culturally-diverse university: boosting happiness and reducing fear. J. Happ. Stud. 20, 1141–1162. 10.1007/s10902-018-9993-z

[B39] Lee Y.-C. Lin Y.-C. Huang C.-L. Fredrickson B. L. (2013). The construct and measurement of peace of mind. J. Happ. Stud. 14, 571–590. 10.1007/s10902-012-9343-5

[B40] Leu J. Wang J. Koo K. (2011). Are positive emotions just as positive across cultures? Emotion 11, 994–999. 10.1037/a0021332 21443338

[B41] Maalouf F. T. Alamiri B. Atweh S. Becker A. E. Cheour M. Darwish H.. (2019). Mental health research in the Arab region: challenges and call for action. Lancet Psychiatry 6, 961–966. 10.1016/S2215-0366(19)30124-5 31327707

[B42] Markus H. R. Kitayama S. (1991). Culture and the self: implications for cognition, emotion, and motivation. Psychol Rev. 98, 224–253. 10.1037/0033-295X.98.2.224

[B43] Mohammed O. Hathloul H. Abdul Rahim Z. (2014). A proposed training program for improving positive thinking and its impact on quality of life and academic achievement among female kindergarten graduates of Al-Jouf University: an experimental study. Al-Jouf J. Soc. Sci. 1, 71–93. 10.12816/0010902

[B44] Peterson C. Seligman M. E. P. (2004). Character Strengths and Virtues: A Handbook and Classification. Washington, DC: American Psychological Association, Oxford University Press.

[B45] Rao M. A. Donaldson S. I. (2015). Expanding opportunities for diversity in positive psychology: an examination of gender, race, and ethnicity. Can. Psychol. 56:271. 10.1037/cap0000036

[B46] Rao M. A. Donaldson S. I. Doiron K. M. (2015). Positive psychology research in the Middle East and North Africa. Middle East J. Positive Psychol. 1, 60–76. Retrieved from https://www.middleeastjournalofpositivepsychology.org/index.php/mejpp/article/view/3333207776

[B47] Rashid T. Al-Haj Baddar M. K. (2019). Positive psychotherapy: clinical and cross-cultural applications of positive psychology, in Positive Psychology in the Middle East/North Africa, eds L. Lambert, and N. Pasha-Zaidi (Cham: Springer), 333–362.

[B48] Rayan A. Ahmad M. (2016). Effectiveness of mindfulness-based interventions on quality of life and positive reappraisal coping among parents of children with autism spectrum disorder. Res. Dev. Disabil. 55, 185–196. 10.1016/j.ridd.2016.04.002 27107368

[B49] Realo A.. (2003). Comparison of public and academic discourses: estonian individualism and collectivism revisited. Cult. Psychol. 9, 47–77. 10.1177/1354067X03009001004

[B50] Realo A. Koido K. Ceulemans E. Allik J. (2002). Three components of individualism. Eur. J. Person. 16, 163–184. 10.1002/per.43725855820

[B51] Saeedi H. Nasab S. H. M. Zadeh A. M. Ebrahimi H. A. (2015). The effectiveness of positive psychology interventions with Islamic approach on quality of life in females with multiple sclerosis. Biomed. Pharmacol. J. 8, 965–970. 10.13005/bpj/848

[B52] Salama-Younes M. Hashim M. (2018). Passion, vitality and life satisfaction for physically active old adults. J. Positive Psychol. 13, 309–319. 10.1080/17439760.2017.1291848

[B53] Salama-Younes M. Massoud W. A.. (2018). Validity and Reliability of Well-being Scales: A Study on Egyptian Physically Active Senior-Aged Adults. *Chronicle of Advances in Positive Health and Well-Being*. 1. Retrieved from https://www.ippanetwork.org/2018/07/27/validity-and-reliability-of-well-being-scales-a-study-on-egyptian-physically-active-senior-aged-adults/

[B54] Salari N. Hosseinian-Far A. Jalali R. Vaisi-Raygani A. Rasoulpoor S. Mohammadi M.. (2020). Prevalence of stress, anxiety, depression among the general population during the COVID-19 pandemic: a systematic review and meta-analysis. Global Health 16:57. 10.1186/s12992-020-00589-w 32631403 PMC7338126

[B55] Seligman M. E. P.. (2011). Flourish: A Visionary New Understanding of Happiness and Well-Being. New York: Free Press.

[B56] Seligman M. E. P. Csikszentmihalyi M. (2000). Positive psychology: an introduction. Am Psychol. 55, 5–14. 10.1037/0003-066X.55.1.5 11392865

[B57] Sin N. L. Lyubomirsky S. (2009). Enhancing well-being and alleviating depressive symptoms with positive psychology interventions: a practice-friendly meta-analysis. J. Clin. Psychol. 65, 467–487. 10.1002/jclp.20593 19301241

[B58] Snyder C. R. Rand K. L. Sigmon D. R. (2002). Hope theory: a member of the positive psychology family, in Handbook of Positive Psychology, eds C. R. Snyder, and S. J. Lopez (New York: Oxford University Press), 257–276.

[B59] Stolarski M. Fieulaine N. Van Beek W. (eds.). (2015). Time Perspective Theory: Review, Research and Application. Cham: Springer International Publishing.

[B60] Thomas J. Raynor M. Bahussain E. (2016). Stress reactivity, depressive symptoms, and mindfulness: a Gulf Arab perspective. Int. Perspect. Psychol. Res. Pract. Consult. 5:156. 10.1037/ipp0000055

[B61] Warsah I.. (2020). Forgiveness viewed from positive psychology and Islam. Islamic Guid. Counsel. J. 3, 108–121. 10.25217/igcj.v3i2.878

[B62] Waters L. Algoe S. B. Dutton J. Emmons R. Fredrickson B. L. Heaphy E.. (2021). Positive psychology in a pandemic: buffering, bolstering, and building mental health. J. Positive Psychol. 16, 1–21. 10.1080/17439760.2021.1871945

[B63] Wulandari I. Megawati F. E. (2020). The role of forgiveness on psychological well-being in adolescents: a review, in 5th ASEAN Conference on Psychology, Counselling, and Humanities (ACPCH 2019) (Atlantis Press), 99–103.

[B64] Younus I.. (2017). The Power of Positive Psychology. Alexandria: Horus International Foundation (in Arabic).

[B65] Zaki H.. (2016). The effectiveness of a training program based on hope in academic procrastination and psychological well-being among education female students. Al-Azhar Univ. Faculty Educ. J. 168, 236–298. 10.21608/JSREP.2016.32074

